# Supplementary material for: Characterization and Optimization of Elastomeric Electrodes for Dielectric Elastomer Artificial Muscles
Source: Materials (Basel). 2020 Dec 4;13(23):5542. doi: 10.3390/ma13235542 (PMC7729933; doi:10.3390/ma13235542)
Supplement: Supplementary file 1 [file materials-13-05542-s001.pdf]

materials

MDPI

Supplementary Material

Characterization and Optimization of Elastomeric Electrodes for Dielectric Elastomer Artificial Muscles

Guangqiang Ma [1], Xiaojun Wu [1,*], Lijin Chen [2], Xin Tong [1] and Weiwei Zhao [2,*]

[1] School of Mechanical and Electrical Engineering, Xi'an University of Architecture and Technology, Xi'an 710055, China; gq.ma@xauat.edu.cn (G.M.); tongxin@xauat.edu.cn (X.T.)
[2] School of Mechanical and Electronic Engineering, Wuhan University of Technology, Wuhan 430070, China; lj.chen@whut.edu.cn (L.C.)
* Correspondence: wuxiaojun@xauat.edu.cn (X.M.) and wzhao@whut.edu.cn (W.Z.)

Received: 2 November 2020; Accepted: 1 December 2020; Published: 4 December 2020

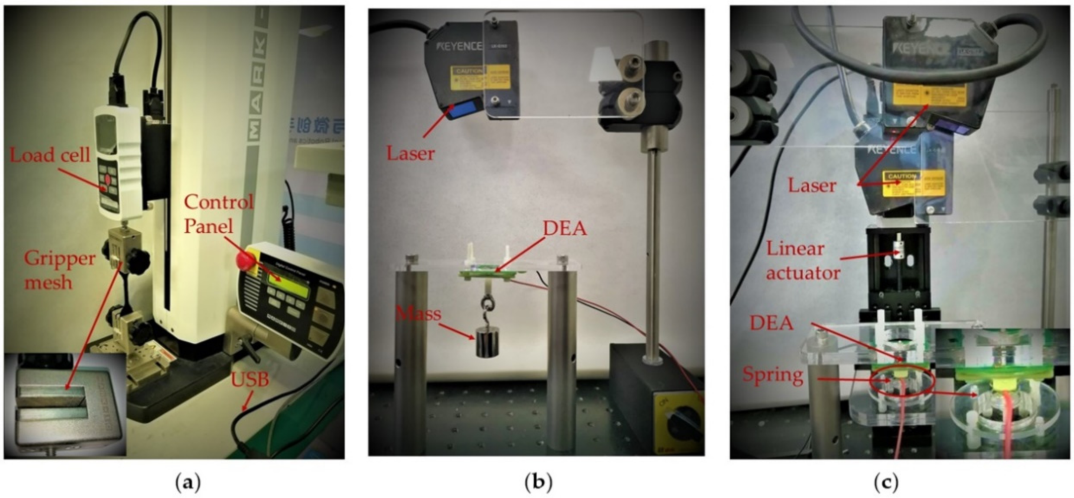

Figure S1. Real setups: (**a**) Tensile test; (**b**) Quasi-static DEA actuation test; (**c**) High-frequency test.

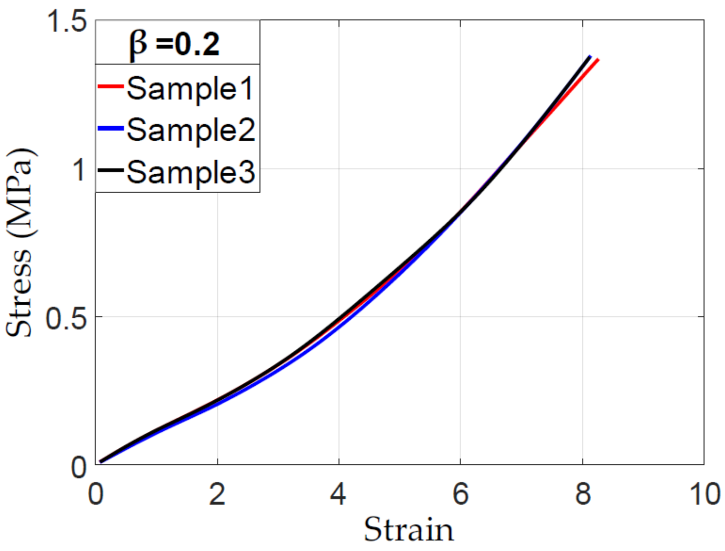

Figure S2. Uniaxial tensile stress–stretch curves from three repeated tensile pull-to-failure tests on the β = 0.2:1 specimen at a strain rate of 49.9 mm/min.

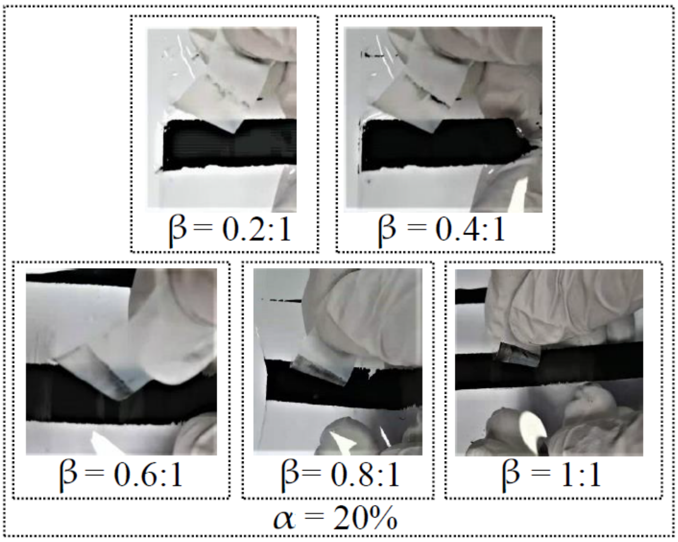

Figure S3. Adhesion experiments at different ratios β.

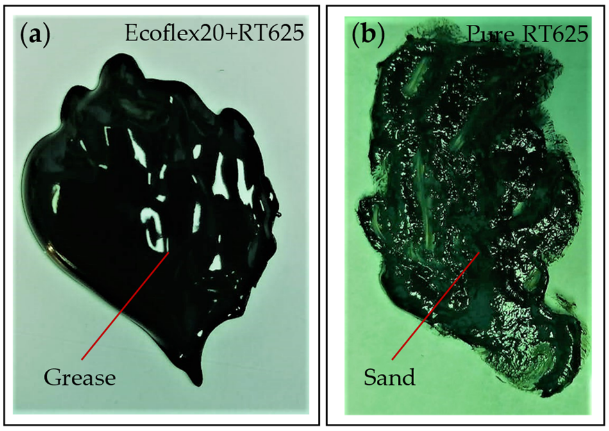

Figure S4. Curing for 2 hours: (**a**) Ecoflex20 + RT625; (**b**) Pure RT625.

© 2020 by the authors. Licensee MDPI, Basel, Switzerland. This article is an open access article distributed under the terms and conditions of the Creative Commons Attribution (CC BY) license (http://creativecommons.org/licenses/by/4.0/).
